# Laparoscopic radiofrequency ablation-assisted enucleation of Xp11.2 translocation renal cell carcinoma: A case report

**DOI:** 10.3892/ol.2014.2267

**Published:** 2014-06-18

**Authors:** LINFENG XU, RONG YANG, WEI WANG, YIFEN ZHANG, WEIDONG GAN

**Affiliations:** Department of Urology, The Affiliated Drum Tower Hospital of the Medical College of Nanjing University, Nanjing, Jiangsu 210008, P.R. China

**Keywords:** renal cell carcinoma, Xp11.2 translocation, radiofrequency, enucleation

## Abstract

The current study presents a case of Xp11.2 translocation renal cell carcinoma (Xp11.2 RCC) in a 30-year-old female. The patient was referred to The Affiliated Drum Tower Hospital of the Medical College of Nanjing University (Nanjing, Jiangsu, China) due to a right renal tumor without evident symptoms, which was found by a routine physical examination. A computed tomography (CT) scan indicated that the mass exhibited cystic and solid components. The patient underwent laparoscopic radiofrequency ablation-assisted enucleation. Immunohistochemistry revealed intense nuclear staining for transcription factor E3 protein in the cancer cells. The patient was diagnosed with Xp11.2 RCC. The urological and radiological outcomes remained satisfactory after >2.5 years of follow-up.

## Introduction

Renal cell carcinoma (RCC) is the most common type of cancer of the adult kidney, accounting for ~3% of all adult malignancies worldwide (1). As a result, RCC is an important cause of cancer morbidity and mortality. Complete surgical excision of the tumor remains the only curative treatment for RCC (2). Radical and partial nephrectomy are alternative treatments with equivalent long-term oncological and renal functional outcomes (3). Xp11.2 translocation RCC (Xp11.2 RCC), a recently classified distinct subtype of RCC, is a rare tumor that typically affects children and young adults. As it is difficult to differentiate Xp11.2 RCCs from conventional RCCs radiologically, the preoperative diagnosis of Xp11.2 RCCs remains challenging, and Xp11.2 RCC is usually treated in the same manner as conventional RCC.

## Case report

**Figure 1:**
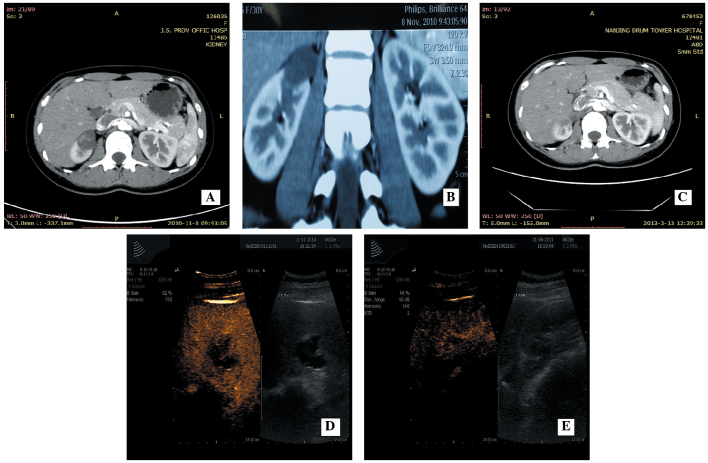
(A) Pre-operative computed tomography (CT) scan showing a 3.0×3.2 cm mass with cystic and solid components at the superior pole of the right kidney. (B) Pre-operative axial CT image. (C) Post-operative CT scan showing no evidence of recurrence. (D) Pre-operative contrast-enhanced ultrasonography showing focal enhancement in the cystic zone. (E) Post-operative contrast-enhanced ultrasonography showing no evidence of recurrence.

A 30-year-old female was referred to The Affiliated Drum Tower Hospital of the Medical College of Nanjing University (Nanjing, Jiangsu, China) due to a right renal mass, found incidentally during a routine physical examination. The patient exhibited no symptoms and the medical history was uneventful. A computed tomography (CT) scan (Fig. 1A and B) showed a 3.0×3.2-cm mass, with cystic and solid components, at the upper pole of the right kidney. Contrast-enhanced ultrasonography showed focal enhancement in the cystic zone, which was different from a renal cyst (Fig. 1D).

**Figure 2:**
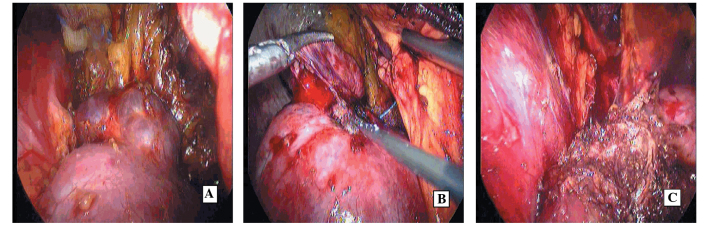
(A) A tumor at the upper pole of the kidney. (B) A cooled electrode was inserted into the kidney between the tumor and normal renal tissue. (C) The tumor bed.

Laparoscopic tumor enucleation (TE) assisted by radiofrequency ablation (RFA) was performed. The renal tumor, which was cystoid and positioned at the upper pole of the right kidney, was exposed via retroperitoneal laparoscopy (Fig. 2A). First, a cooled electrode was inserted in the kidney between the tumor and normal renal tissue under ultrasound guidance (Fig. 2B). The electrode was internally cooled with a peristaltic pump circulating chilled water, and the tip temperature of the electrode was kept ~12°C. The electrical power was elevated to 100 W. RFA lasted for 10 min. Next, the ablation was performed again on an area 2 cm away from the first electrode. Finally, the tumor was removed along the pseudocapsule (Fig. 2C).

Gross examination showed a cystoid tumor of 3.0 cm in diameter, with solid tissue and septa inside. The hematoxylin and eosin-stained histopathological specimen revealed predominant tumor cells showing papillary architectures with calcification. A fibrous capsule surrounded the tumor. The pathological stage was classified as pT1aNxM0. Immunochemistry for transcription factor E3 (TFE3) showed intense nuclear staining in the majority of the tumor cells.

Subsequent to 2.5 years of follow-up, the patient showed no evidence of disease on contrast-enhanced CT and ultrasonography (Fig. 1C and E).

## Discussion

Xp11.2 RCCs have been present in the World Health Organization classification of kidney tumors for nine years (4). Xp11.2 RCCs are relatively rare tumors that typically occur in children and young adults. Approximately one-third of pediatric RCCs are estimated to be Xp11.2 RCCs associated with TFE3 gene fusion. The incidence in adults has recently been evaluated as 1.5% of all types of RCCs (5). The exact frequency of Xp11.2 RCCs is actually underestimated in patients >40 years old, as its histological features often mimic clear cell RCCs or papillary RCCs.

Xp11.2 RCCs can present with macroscopic hematuria, flank pain, colic, masses and even metastatic symptoms first. It is more common for patients with Xp11.2 RCCs to exhibit symptoms than those with clear cell RCCs. The current case presented with only a right renal mass, without any symptoms.

Xp11.2 RCCs originate from the renal medulla, manifesting as cystic or solid masses. Zhu *et al* (6) suggested that the density of Xp11.2 RCCs was greater than that of the normal renal cortex and medulla on unenhanced CT. The enhancement was higher than that observed in the renal medulla during the cortical and medullary phases, but lower than that in the normal renal medulla during the delayed phase. However, it is not uncommon to manifest multilocular cystic RCC-like CT images for Xp11.2 RCCs (7,8). This case appeared as a cystoid mass that contained low attenuating necrotic or hemorrhagic foci on unenhanced images and a well-defined mass with focal enhanced solid portions on enhanced images.

As Xp11.2 RCCs resemble conventional RCCs radiologically, the pre-operative diagnosis of Xp11.2 RCCs remains challenging, and this type of RCC is usually treated in the same manner as conventional RCC.

At present, the treatment for RCCs remains as surgical excision. Radical nephrectomy and partial nephrectomy (PN) are alternative treatments with equivalent long-term oncological and renal functional outcomes. The enucleation technique has recently been developed following attempts to further spare the renal parenchyma. TE has equivalent oncological outcomes to partial nephrectomy, particularly for small renal masses (9). TE has been associated with a 16% adverse event rate, and of those events, only 3% required re-intervention (10). Minervini *et al* (11) reported that three out of 164 (1.8%) patients exhibited local recurrence; one (0.6%) presented with true local recurrence at the enucleation site detected at 35 months post-surgery, while two presented with kidney recurrence elsewhere that was associated with concurrent systemic metastases diagnosed at 16 and 13 months post-surgery.

RFA, as a minimally invasive treatment, can assist surgical procedures. A needle is introduced into the tumor and produces an increase in temperature high enough to destroy the tumor cells, while transmitting minimal collateral damage to the surrounding renal parenchyma (12). Prior to TE, radiofrequency coagulation can be used to make the surrounding parenchymal vessel occlusive via a cooled electrode inserted in the kidney between the tumor and normal renal tissue. TE is relatively bloodless, obviating the requirement for hilar clamping (13), and the ablation ensures that the surviving tumor cells are killed in the tumor bed. So RFA-assisted TE can protect the renal unit whilst eliminating residual tumor cells. To the best of our knowledge, the present case is the first Xp11.2 RCC treated with laparoscopic RFA-assisted enucleation.

The clinical course of this tumor type is heterogeneous. While certain cases behave indolently, such as the present case, other cases may behave quite aggressively (14). An age of >50 years may be associated with a poor prognosis (15).

The urological and radiological outcomes of this case were satisfactory, which may be attributed to the tumor dimension and the initial presentation. Although this type of renal cancer is prone to lymph node metastasis prior to surgical intervention, a few XP11.2 RCCs treated with partial nephrectomy have been found with no recurrence or metastasis in the limited studies available. In the studies by Argani *et al* (1/28 cases; 6-month follow-up) (16) and Komai *et al* (2/7 cases; 96- and 132-month follow-up, respectively) (17), Xp11.2 RCC patients with small tumors (<4 cm) and no symptoms were shown to have usually favorable outcomes subsequent to PN. More time is required to further observe this type of RCC, which may belong to a special subtype of Xp11.2 RCCs.

In conclusion, laparoscopic RFA-assisted enucleation may be an effective method for Xp11.2 RCC patients with small tumors (<4 cm) and no symptoms. The patient in the present study had a favorable clinical course. More data and longer follow-up times are required to determine the optimal treatment methods and outcomes for this type. The rare and sporadic nature of the cases restricts currently restricts multi-sample research.
